# Supplementary material for: Design, Synthesis, and Tribological Behavior of an Eco-Friendly Methylbenzotriazole-Amide Derivative
Source: Int J Mol Sci. 2025 Jan 27;26(3):1112. doi: 10.3390/ijms26031112 (PMC11818913; doi:10.3390/ijms26031112)
Supplement: Supplementary file 1 [file ijms-26-01112-s001.zip › ijms-3430763-supplementary.pdf]

Design, Synthesis, and Tribological Behavior of An Eco-friendly Methylbenzotriazole-Amide Derivative

Supporting information

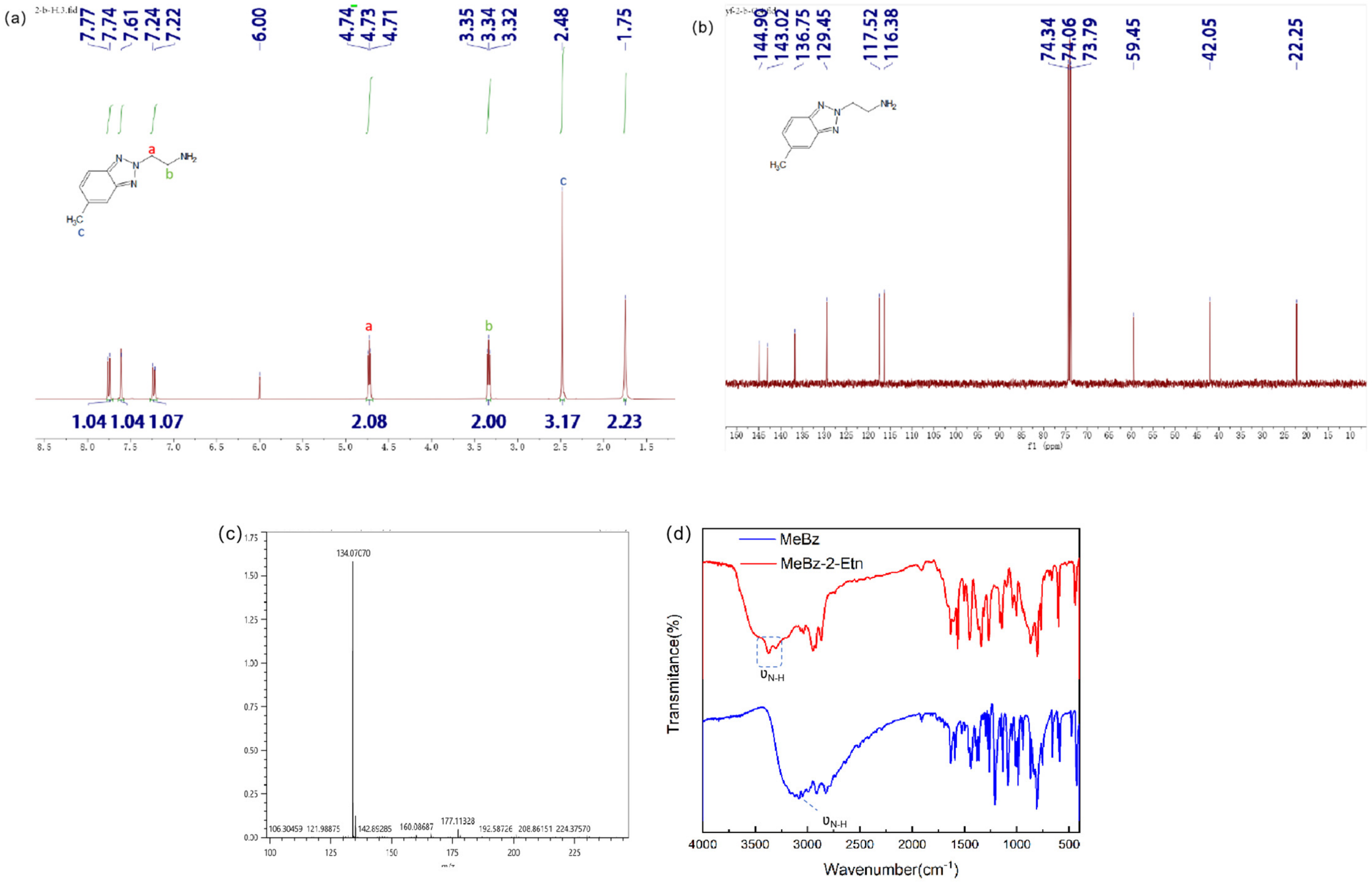

Figure S1 The characterizations of MeBz-2-En: (a) ^{1}H NMR, (b) ^{13}C NMR, (c) HR-MS and (d) FT-IR.

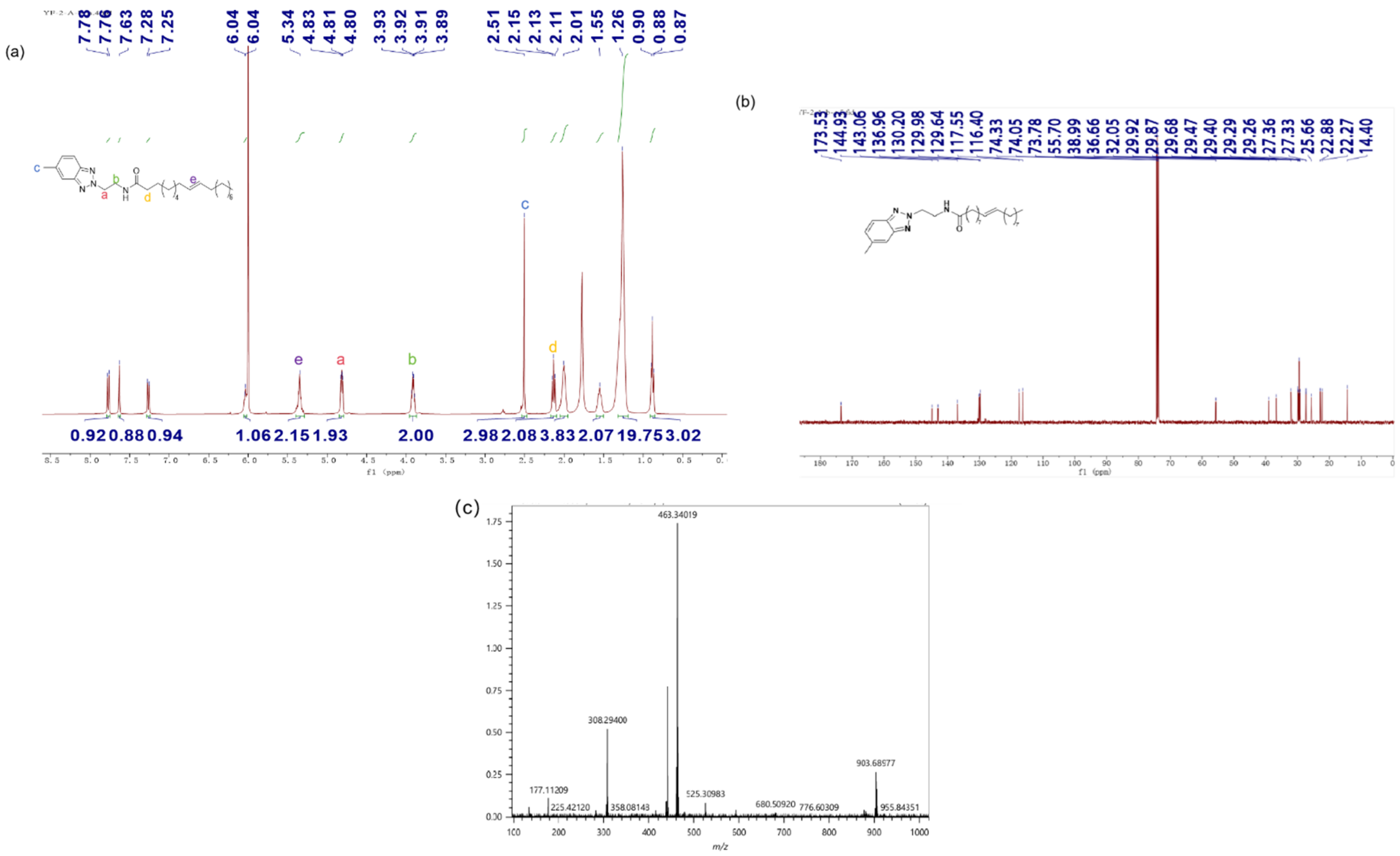

Figure S2 The characterizations of MeBz-2-C18: (a) ^{1}H NMR, (b) ^{13}C NMR and (c) HR-MS.
